# IPINeT Ped-unPAD Study: Goals, Design, and Preliminary Results

**DOI:** 10.3390/jcm13154321

**Published:** 2024-07-24

**Authors:** Mayla Sgrulletti, Lucia Augusta Baselli, Riccardo Castagnoli, Elisabetta Del Duca, Simona Graziani, Giusella Maria Francesca Moscato, Silvia Di Cesare, Gigliola Di Matteo, Cristina Cifaldi, Martina Rossano, Claudia Ballerini, Alfonso Piciocchi, Amelia Licari, Gian Luigi Marseglia, Rita Consolini, Viviana Moschese

**Affiliations:** 1 Pediatric Immunopathology and Allergology Unit, Policlinico Tor Vergata, University of Rome Tor Vergata, 00133 Rome, Italy; maylasg@gmail.com (M.S.); elisabetta.delduca@ptvonline.it (E.D.D.); simona.graziani@infinito.it (S.G.); gius_va@virgilio.it (G.M.F.M.); 2 PhD Program in Immunology, Molecular Medicine and Applied Biotechnology, University of Rome Tor Vergata, 00133 Rome, Italy; 3 Pediatric Immunorheumatology Unit, Fondazione IRCCS Ca’ Granda Ospedale Maggiore Policlinico, 20122 Milan, Italy; lucia.baselli@policlinico.mi.it (L.A.B.); martina.rossano@policlinico.mi.it (M.R.); 4 Pediatric Unit, Department of Clinical, Surgical, Diagnostic, and Pediatric Sciences, University of Pavia, 27100 Pavia, Italy; riccardo.castagnoli@unipv.it (R.C.); a.licari@smatteo.pv.it (A.L.); gl.marseglia@smatteo.pv.it (G.L.M.); 5 Pediatric Clinic, Fondazione IRCCS Policlinico San Matteo, 27100 Pavia, Italy; 6 Department of System Medicine, University of Rome Tor Vergata, 00133 Rome, Italy; di.matteo@med.uniroma2.it (G.D.M.); cristina.cifaldi@gmail.com (C.C.); 7 Unit of Clinical Immunology and Vaccinology, IRCCS Bambino Gesù Children’s Hospital, 00165 Rome, Italy; di.cesare@med.uniroma2.it; 8 Primary Immunodeficiency Research Unit, IRCCS Bambino Gesù Children’s Hospital, 00165 Rome, Italy; 9 Università degli Studi di Milano, 20122 Milan, Italy; claudiaballerini28@gmail.com; 10 GIMEMA Data Center, Fondazione GIMEMA Franco Mandelli Onlus, 00182 Rome, Italy; a.piciocchi@gimema.it; 11 Section of Clinical and Laboratory Immunology, Pediatrics Unit, Department of Clinical and Experimental Medicine, University of Pisa, 56126 Pisa, Italy; rita.consolini@med.unipi.it

**Keywords:** children, common variable immunodeficiency, inborn errors of immunity, primary antibody deficiency, transient hypogammaglobulinemia of infancy, unclassified primary antibody deficiency

## Abstract

**Background:** An unclassified primary antibody deficiency (unPAD) is a widely heterogeneous clinical entity, recently identified within the spectrum of Inborn Errors of Immunity (IEIs). Since unPAD has been traditionally considered as a mild condition, it has incorrectly received little attention, resulting in the paucity of extensive and comparable studies describing its natural history. To address the gaps in characterizing, understanding, and managing pediatric unPAD patients, the Italian Primary Immunodeficiency Network (IPINet) Ped-unPAD study has recently been launched. **Methods:** Seventeen IPINeT Centers have expressed interest to participate, and data collection is still on-going. Hereby, we anticipate preliminary key issues emerging from the first 110 enrolled patients, attending three IPINet Centers. **Results:** A proportion of unPAD patients have experienced a severe infectious phenotype, which required hospitalization in a quarter of patients and antibiotic prophylaxis or Immunoglobulin Replacement Therapy in approximately 10% of patients. In this partial cohort, a mean follow-up (FU) of 5 years confirmed unPAD diagnosis in fifty percent of cases, with the remaining being reclassified as the Transient Hypogammaglobulinemia of Infancy (25%) and other IEIs (25%), such as a Common Variable Immunodeficiency, Selective IgA deficiency, Selective IgM deficiency, and IgG3 subclass deficiency. **Conclusions:** Despite a phenotype overlap at diagnosis, clinicians should be aware that unPAD is a mutable condition that deserves comprehensive evaluation and long-term monitoring to dissect the final diagnosis for optimal treatment.

## 1. Background

An unclassified primary antibody deficiency (unPAD) has recently been included in the European Society for Immunodeficiencies’ (ESID) working definitions for the clinical diagnosis of Inborn Errors of Immunity (IEI) as a distinct nosologic entity. The ESID criteria for a possible unPAD diagnosis are based on the presence of marked hypogammaglobulinemia (low levels of one or more immunoglobulins—IgG, IgG subclasses, IgA, or IgM) and/or evidence of an impaired IgG antibody response to vaccination. Additionally, at least one of the following clinical features must be documented: (a) recurrent/severe bacterial infections, (b) autoimmune manifestations (particularly cytopenias), (c) polyclonal lymphoproliferation, or (d) a positive family history for an IEI. Of note, secondary causes of hypogammaglobulinemia as well as T-cell immunodeficiency need to be excluded [1].

UnPAD prevalence in the general population is currently unknown. However, according to the latest ESID registry update, an “unclassified antibody deficiency” represents 15.7% of Primary Antibody Disorders (PADs), accounting for about 2000 patients suffering from this condition [2]. Furthermore, this number largely doubles with the inclusion of patients suffering from an “isolated IgG subclass deficiency”, “IgA deficiency with IgG subclass deficiency”, and a “specific antibody deficiency”. With this reasoning, the number of unPAD patients may be comparable to that of Common Variable Immunodeficiency (CVID) patients (over 5000 in the ESID registry), with an estimated prevalence in the general population of 1:10,000–50,000 [2]. UnPAD patients show a highly variable clinical spectrum [3]. Notably, some patients may be completely asymptomatic at the first examination and develop clinical manifestations over time [4]. In symptomatic patients, recurrent infections, mostly involving the upper respiratory tract (URTI), represent the main clinical finding. Although these patients mainly suffer from recurrent mild and “common for age” infections, which are responsive to conventional treatment, more severe infectious diseases such as lower respiratory tract and urinary tract infections have also been reported. Unexpected or opportunistic pathogens are rarely involved [5,6,7]. Moreover, as for other PADs, a higher frequency of asthma and atopy has also been detected [8]. UnPAD patients show highly variable laboratory features, too. Ig mean values appear to be higher than those observed in CVID patients, and specific antibody responses, mainly to the pneumococcal vaccine, may be variably impaired. Although, in some case series, unPAD patients’ B-cell subsets match healthy control values, no substantial difference in B-lymphocyte subset distribution has been recently detected in a cohort of unPAD patients receiving Ig replacement therapy compared to CVID patients [9]. Due to the common opinion that unPAD patients suffer from a milder infectious phenotype and less compromised Ig levels than CVID, they may remain untested and unrecognized for a long time, with an underestimated risk of organ damage. For the same reasons, data on the frequency of their autoimmune and lymphoproliferative complications are extremely scarce, as well as on their clinical course and outcome.

UnPAD is sometimes a “comfort diagnosis” due to its wide heterogeneity and, at times, transient condition. Some unPAD patients may over time develop more defined PADs, such as an IgA deficiency, IgM deficiency, and CVID, and be reclassified. Some others, initially identified as UnPAD, may later normalize their IgG levels and receive an a posteriori diagnosis of Transient Hypogammaglobulinemia of Infancy (THI). Few studies on the clinical course and outcome of these patients are currently available. Despite Ig, normalization occurs more frequently within the first 4 years of age and, mostly in the first 24 months [10], the time of recovery may be delayed until the third or fourth decade of life [4,11]. Usually, a less severe clinical and immunological phenotype and a milder course of the disease over time have been associated with IgG normalization, in contrast to a serious clinical picture (i.e., severe infections and/or autoimmune manifestations) as well as an impairment of the class-switched memory B-cell compartment with the persistence of hypogammaglobulinemia [4,10,12].

Data regarding the treatment of unPAD patients are extremely scarce, especially in the pediatric age [13]. The current assumption is that unPAD patients do not require specific therapeutic interventions due to their mild clinical picture and moderate hypogammaglobulinemia [14]. However, in some cases, they present with severe infectious and non-infectious manifestations, requiring appropriate therapeutic management [15]. In addition, even a mild phenotype characterized by the recurrence of “normal, not complicated infections” (i.e., responding to conventional treatments and not caused by an unexpected or opportunistic pathogen) can negatively impact quality of life due to impaired social engagement (e.g., school or work absenteeism) and an increased economic burden on healthcare.

The mainstays of the management of non-mild unPAD patients have been derived from therapeutic interventions of congenital agammaglobulinemia and CVID. They include antimicrobial prophylaxis, immunoglobulin replacement therapy (IRT), vaccinations, treatment for non-infectious manifestations, physiotherapy/airway treatment, and psychological support [16]. However, as mentioned above, the clinical and immunological variability of unPAD makes it challenging to define a “one-fits-all” approach, and a therapeutic plan tailored for each patient is mandatory.

To fill the gaps in the characterization, understanding, and management of unPAD patients and allow a better comprehension of the natural history of the disease, a Ped-unPAD study has been recently implemented within the framework of the Italian Primary Immunodeficiency Network (IPINet).

## 2. Methods

### 2.1. Ped-unPAD Study Goals and Study Design

Ped-unPAD study is a national multicenter prospective and retrospective observational cohort study which aims to (I) evaluate the real incidence of unPAD among all types of PADs and define the minimal prevalence in our national setting, (II) define its natural history in terms of disease onset, progression, and treatment response, and (III) identify predictive/prognostic markers of clinical outcome. Moreover, the detailed description of the clinical–immunological phenotype at diagnosis and at follow-up together with gene data might allow a better understanding of disease expressivity and clinical outcome. A list of known candidate genes associated with PADs is reported in Table 1.

We will evaluate the time span from symptoms onset to clinical diagnosis in order to quantify the median diagnostic delay for unPAD condition in the Italian scenario. Last but not least, patients’ quality of life will be investigated. Research interests of Ped-unPAD study are reported in Table 2**.**

*Inclusion Criteria* include the following:

Pediatric (1–18 years) female or male patients with at least 1 of the following clinical criteria:

- Absence of symptoms.
- Recurrent bacterial infections (>6 upper tract respiratory infections and/or >3 acute otitis media and/or >1 acute sinusitis and/or >1 bronchopneumonia in one year) or severe bacterial infections (abscesses, sepsis, meningitis, or osteomyelitis).
- Autoimmune diseases (i.e., cytopenia, thyroiditis, diabetes, SLE, alopecia, psoriasis, or Addison Disease).
- Non-malignant lymphoid proliferation.
- Positive family history for IEI.

Plus at least one of the following laboratory criteria:

- IgG values < 2 SD of the normal levels for the age and/or normal or reduced IgG1, IgG2, and IgG3 values and/or normal or reduced IgA values and/or normal or reduced IgM values with impaired or normal specific antibody response to vaccines.
- Combined IgA, IgM, and IgG subclass defect, with impaired or normal specific antibody response to vaccines.

AND:

- No defects of T-cell compartment.

The following are the *Exclusion Criteria*:

- Refusal of parents or legal guardians to sign written informed consent.
- Documented secondary causes of hypogammaglobulinemia due to infections, hematologic/neoplastic or iatrogenic conditions, genetic syndromes, chromosomal abnormalities, protein-losing enteropathy and/or nephropathy, and thymoma.
- Diagnosis of other IEI according to ESID criteria.

### 2.2. Recruiting Measures, Data Collection, and Storage

Ped-unPAD study is open to all IPINeT Centers that wish to participate and have the approval of their Local Ethical Committees. Data are collected using REDCap (Research Electronic Data Capture) tools hosted at the University of Rome Tor Vergata [18,19].

REDCap is a secure, web-based software platform designed to support data capture for research studies. It offers an intuitive interface for validated data entry, audit trails for tracking data manipulation and export processes, automated export procedures for seamless data downloads to common statistical packages, and features for data integration and interoperability with external sources (https://www.project-redcap.org, accessed on 19 July 2024).

An Electronic-Case Report Form (e-CRF), specifically implemented on REDCap web application, will be filled out by Clinicians of IPINeT Centers for each patient matching the inclusion criteria at diagnosis and every 12 months.

REDCap service provides compliance with regulations about privacy protection including new European GDPR (n. 679/2016). All data are collected anonymously, as to local privacy policies. To maintain anonymity, patients are identified only by the initials, with the date of birth representing the only sensitive data recorded in the e-CRF. Written informed consent for data collection is obtained from each patient or their legal guardians by the treating physician. Information entered into e-CRFs will derive from patient’s paper and electronic medical records and local research databases.

The information to be collected will include the following: demographic data, family history, consanguinity, clinical and immunological features, genetic analysis, instrumental investigations, and both past/current treatments as well as quality of life evaluation (Table 3).

### 2.3. Sample Size and Statistical Analysis

To provide a comprehensive description of unPAD patients, we aim to gather data from as many individuals as possible. Based on the number of registered unPAD patients in the ESID online Registry, we plan to enroll a minimum of 1000 patients. This sample size allows us to analyze demographic, clinical, and immunological characteristics both at presentation and throughout follow-up, as well as to assess the risk of complications in potentially significant subgroups. Due to the exploratory nature of this study, no power calculation was carried out to ascertain the sample size.

Patients’ characteristics are summarized using cross-tabulations for categorical variables, quantiles (e.g., median) for ordinal variables, and standard measures of central tendency and dispersion (e.g., mean, standard deviation) for continuous variables.

Differences among groups are assessed using univariate analysis by non-parametric tests, such as the Chi-Squared and Fisher Exact tests for categorical variables or response rates, and the Mann–Whitney and Kruskal–Wallis tests for continuous variables.

Logistic regression models are employed in both univariate and multivariate analyses to determine if clinical and biological parameters are associated with diagnosis. Results of the logistic regression models are reported as Odds Ratios (OR) with 95% Confidence Intervals.

All tests are two-sided, with *p*-values < 0.05 indicating statistical significance, and confidence intervals calculated at the 95% level. All analyses are conducted using R software (R Core Team, 2022, release di R 4.3.2) [20].

## 3. Preliminary Results

One hundred and sixty-one pediatric patients, enrolled at the Pediatric Immunopathology and Allergology Unit/Regional Referral Center for IEIs at Policlinico Tor Vergata in Rome, the Pediatric Clinic of Fondazione IRCCS Policlinico San Matteo in Pavia, Italy, and the Department of Pediatrics of Fondazione IRCCS Ca’ Granda Ospedale Maggiore Policlinico in Milan, Italy, have been registered on the platform. Fourteen additional centers are willing to participate, and data collection is still on-going. Since follow-up data are available for 110/161 patients, a preliminary analysis has been focused on this cohort.

A total of 110 pediatric patients (72 males, 65%, and 38 females, 35%) initially diagnosed as unPAD entered in this study. The median age at diagnosis was 4 years (range 1–17 years). Among 14/110 (13%) patients with a positive family history for IEIs, two had parental consanguinity. Also, 13/110 (12%) and 28/110 (25%) patients had a positive family history of autoimmunity and allergies, respectively.

One hundred and four patients (104/110, 94%; 55/59, 93% < 4 years and 49/51, 96% > 4 years) were symptomatic at diagnosis with a median age at clinical onset of 24 months (range 1 months–17 years). As reported in Table 4, recurrent infections—mainly involving the respiratory, gastrointestinal, and urinary tracts—represent the most common clinical manifestations (84/104, 80%). Before reaching the diagnosis, 26/84 (31%) and 5/84 (6%) patients experienced one or more episodes of pneumonia and skin abscesses, respectively. Recurrent respiratory tract infections (RRTI), pneumonia, and skin abscesses were more frequently observed in patients > 4 years of age (RRTI 8/46, 17% < 4 years of age cohort vs. 18/38, 47% > 4 years of age cohort, *p*-value 0.04; pneumonia 9/46, 19% < 4 years of age cohort vs. 17/38, 45% > 4 years of age cohort, *p*-value 0.018; skin abscesses 0/46, 0% < 4 years of age cohort vs. 5/38, 13% > 4 years of age cohort, *p*-value 0.016). Of note, in two patients, belonging to the >4 years of age cohort, an episode of meningitis (due to pneumococcus and varicella zoster virus, respectively) has been reported. Allergies and autoimmune diseases are present in 37/104 patients (36%) and 9/104 patients (9%), respectively. Allergies result as more frequent in the older group (14/55, 25% < 4 years vs. 23/49, 47% > 4 years, *p* value 0.0257) as well as for autoimmunity, whose frequency seems to increase with age, although statistical significance has not been reached. In one patient, bronchiectasis was detected at diagnosis and four patients (4%) experienced benign lymphoproliferation. Twenty-eight patients (25%) were hospitalized—an average of 2 times—before diagnosis with no correlation with age at diagnosis. However, in the context of hospitalization due to pneumonia—which represented the common condition (10/28, 36%)—a higher frequency was observed in the older cohort (2/15, 13% < 4 years of age cohort vs. 8/13, 61% > 4 years of age cohort, *p*-value 0.016). Due to a more severe clinical picture, 3/110 (3%) and 7/110 (6%) children started IgG replacement therapy and antibiotic prophylaxis, respectively.

At diagnosis, UnPAD patients showed a widely heterogeneous immunological profile. In detail, 74%, 47%, and 54% patients had isolated or combined IgG, IgA, and IgM defects (compared with age-appropriate values), respectively. Younger patients were more likely to exhibit IgA deficiency, isolated or combined with other Ig isotype defect (37/59, 63% < 4 years vs. 15/51, 29% > 4 years, *p* value 0.0006). Fifty-nine children (70%) presented IgG subclass deficiency. An impaired specific antibody response to tetanus and pneumococcus vaccines was observed in 8/54 (15%) and 13/43 (30%), respectively, with no correlation with age at diagnosis.

The overall cohort had a normal standard immunophenotype analysis. However, extended B-cell immunophenotyping available for 44/110 patients showed that 22/44 (50%) and 4/44 (9%) had reduced switched memory B cells and IgM memory B cells, respectively.

**Figure 1:**
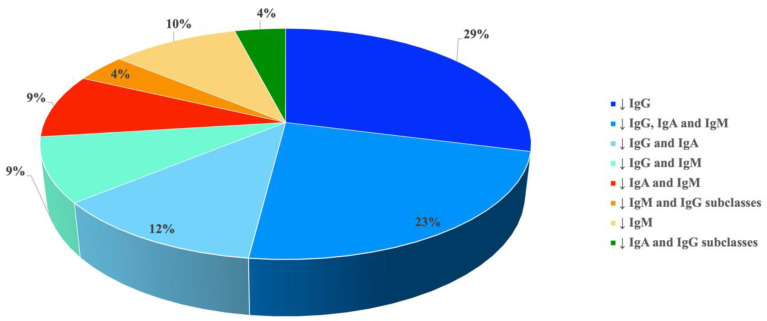
Patient stratification according to reduced Ig isotype(s)/IgG subclasses.

A detailed representation of patient’s stratification according to reduced Ig isotype(s)/IgG subclasses is reported in Figure 1.

**Figure 2:**
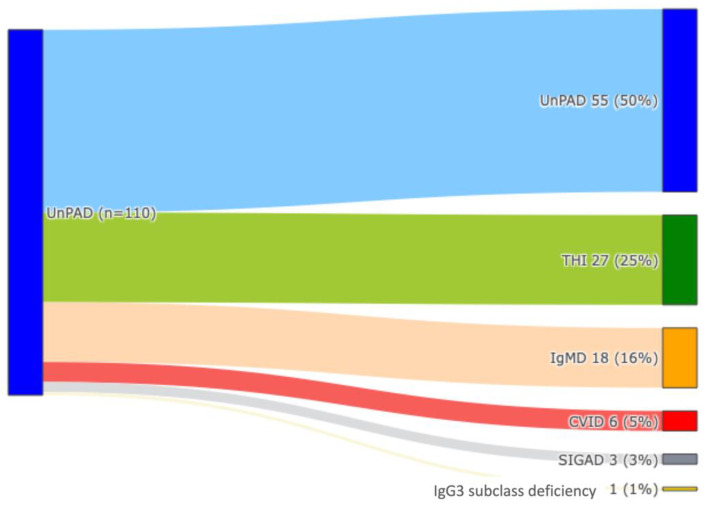
Diagnostic reclassification of 110 unPAD patients at last FU: unPAD (clear blue), THI (green), IgMD (orange), CVID (red), SIGAD (grey), IgG subclass deficiency (yellow).

As per standard practice, all 110 patients received regular clinical and immunological follow-up every 6 months for a mean time of 5 years (range 1–33 years). As shown in Figure 2, at the last follow-up, 27/110 (25%) patients reached age-appropriate Ig values and entered in the category of THI, whereas 83 patients (75%) showed persistence of an IEI condition. In detail, an unPAD diagnosis was made in 55/110 (50%), whereas 18/110 (16%), 6/110 (5%), 3/110 (3%), and 1/110 (1%) patients developed a selective IgM deficiency, CVID, selective IgA deficiency, and IgG3 subclass deficiency, respectively.

Table 5 compares the clinical and immunological features at diagnosis between patients with persistent IEI (n = 83) and those with THI (n = 27) at last follow-up.

Only one of the 6 patients who were asymptomatic at diagnosis normalized his Ig levels, confirming the diagnosis of THI. The other 5 patients were reclassified as unPAD (3 patients), IgM deficiency (1 patient), and CVID (1 patient). Patients with persistent IEI were mainly diagnosed at a later age (over 4 years old) compared to those with a final diagnosis of THI (45/83, 54% IEI vs. 6/27, 22% THI, *p* value 0.004). Additionally, a history of RRTI and pneumonia were significantly more common in the group with persistent IEI (32/61, 52% IEI patients vs. 6/23, 26% THI patients, *p* value 0.03; 22/61, 36% IEI patients vs. 4/23, 17% THI patients, *p* value 0.04, respectively). Although we detected no significant difference in the hospitalization rate at diagnosis between the two groups, hospitalization due to serious infections (i.e., pneumonia, meningitis, cellulitis, and urinary tract infections/UTI) was statistically more frequent in patients with a final diagnosis of IEI (13/20, 65% IEI patients vs. 1/8, 12% THI patients, *p* value 0.03). Again, even though a small cohort of patients with a final diagnosis of THI experienced pneumonia at diagnosis, this condition was responsible for hospitalization only in patients with a final diagnosis of IEI (10/20, 50% IEI patients vs. 0/8, 0% THI patients, *p* value 0.02). Of note, all patients with previous hospitalizations due to pneumonia, meningitis, and UTI received a diagnosis of persistent unPAD at the last FU. Moreover, all ten children who, at diagnosis, required IgG replacement therapy or antibiotic prophylaxis belonged to the persistent IEI patients group (4 and 6 patients with a final diagnosis of CVID and unPAD, respectively). No significant clinical difference at diagnosis was detected in the 6 patients with a final diagnosis of CVID compared to the 55 patients with a final unPAD diagnosis.

**Figure 3:**
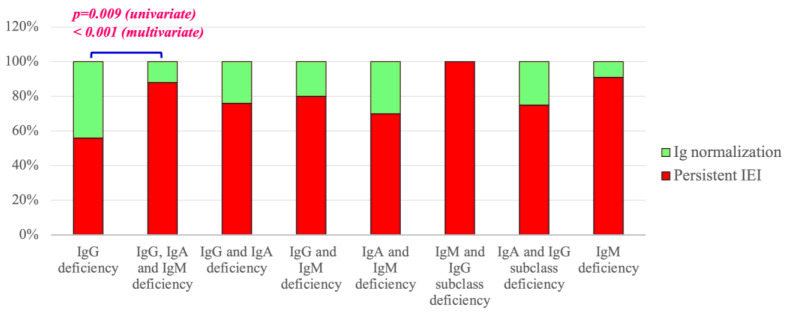
IEI evolution according to reduced Ig isotype(s)/IgG subclasses at diagnosis.

Looking at immunological findings at diagnosis, as detailed in Table 5 and Figure 3, isolated or combined IgM deficiency, combined deficiency of IgA and IgM, combined IgG, IgA and IgM defect, low anti-PCP antibody response, and low switched memory B cells were associated with a persistent IEI condition. In contrast, the sole IgG defect was significantly associated with final Ig normalization (18/83, 22% IEI patients vs. 14/27, 52% THI patients, *p* value < 0.001).

## 4. Discussion

The patient registries and study protocols represent essential tools to allow a better comprehension of rare pathological conditions [21]. As for other rare diseases, IEIs represent a real challenge for clinicians due to their intrinsic nature of rarity, heterogeneity, and complexity, that could result in a considerable diagnostic delay, which is of concern given the crucial role of early intervention in preventing the development of severe complications and organ dysfunction [22]. UnPAD represent a relatively recent new clinical entity which is widely heterogeneous, which, due to both its recent identification and its traditionally considered mild clinical–immunological picture, has hardly received adequate attention from the scientific community with consequent paucity of research investigations in the international literature [13]. Currently, no peculiar features have been identified to allow transient hypogammaglobulinemia to be distinguished from other forms of hypogammaglobulinemia at the time of diagnosis, which would be relevant to adjust timely and appropriate monitoring and treatment regimens. In a recent paper, by investigating a cohort of 23 unPAD patients monitored for a mean time of 14 years, we showed how unPAD diagnosis can change over time. As per the last follow-up, 56% of patients exhibited a persistent IEI. Within this group, unPAD remained the most frequent diagnosis affecting 30% of patients, whereas 13%, 9%, and 4% developed common variable immunodeficiency, selective IgA deficiency, and isolated IgM deficiency, respectively [4]. Based on these results and the awareness that the clinical and immunological picture of these patients can be mildly impaired at the time of first examination, we highlight that long-term monitoring with regular steps of clinical review is crucial for the patient’s outcome and the possible achievement of a definitive diagnosis.

In this regard, the implementation of the IPINet Ped-unPAD study (as mentioned in the Material and Methods section) will prove useful in the elaboration of a work-up process to decrease the diagnostic delay and optimize the clinical care of unPAD patients.

Since the Ped-unPAD study represents a rather recent initiative within IPINeT, data collection is still on-going. In this paper, we present the data obtained from the analysis of the initial 110 unPAD children enrolled, attending three IPINet Centers. Although their limited number is not representative of the entire Italian unPAD cohort, the preliminary data draw attention to interesting clues.

A small proportion of patients were asymptomatic at diagnosis; although this phenomenon remains unclear, it has been argued that epigenetic, immunological, and environmental factors might have a compensatory role for a certain time, being potential triggers for the subsequent development of clinical signs. According to previous reports [5,6,7], recurrent respiratory infections were the predominant manifestations of the cohort at the time of diagnosis. However, a proportion of unPAD patients experienced a severe infectious phenotype, which required hospitalization in a quarter of patients, due mainly to one or more episodes of pneumonia. Interestingly, being > 4 years of age was associated to severe infections and pneumonia-related hospitalizations. Other infective causes such as skin abscesses and meningitis, due to *S. Pneumonia* and *Varicella Zoster Virus,* were less frequently observed. Thirty-six percent of unPAD patients suffered from allergies, whereas autoimmune manifestations were less common (9/104, 9%). Allergies and autoimmunity seem to increase with age, although statistical significance was not reached in the case of autoimmune diseases. This is consistent with previous studies on PAD patients showing how these patients might develop immune dysregulatory features over time, mainly in early and late adulthood [3,23].

UnPAD patients show a high variability, also in terms of laboratory features. Although Ig levels appear to be higher than in CVID patients, a specific antibody response, mainly to the pneumococcal vaccine, may be variably impaired. It has been reported that some unPAD patients may exhibit B cell abnormalities similar to those observed in individuals with various antibody deficiencies, mostly CVID [9].

The analysis of the extended B cell immunophenotyping in 44/110 patients of our cohort confirmed these results. Indeed, 50% and 9% of patients showed a reduction in switched memory B cells and IgM memory B cells, respectively. Additionally, an inadequate antibody response to tetanus and pneumococcal vaccinations was observed in 8/54 (15%) and 13/43 (30%) patients, respectively. The results obtained by stratifying the patients, according to the Ig isotype/Ig subclass levels, markedly demonstrated the wide laboratory heterogeneity of the unPAD condition, supporting its role of “basket diagnosis”.

The treatment of UnPAD patients has been poorly discussed, especially in the pediatric age [13]. The widespread opinion is that these patients have a mild clinical phenotype because of their moderate decrease in immunoglobulin levels, thus requiring no significant therapeutic interventions [14]. However, our data demonstrated that unPAD children may present with a wide heterogeneous clinical phenotype, also including severe infectious and non-infectious manifestations [15]. Approximately 10% of our patients received antibiotic prophylaxis or IRT at diagnosis, with improved clinical control. Currently, no guidelines exist for antibiotic prophylaxis and IRT in unPAD patients, especially in the pediatric age [16]. Considering the susceptibility of these patients to recurrent and severe bacterial infections, we recommend a prompt clinical evaluation and carrying out a broad-spectrum antimicrobial therapy in the case of fever and manifestations of an acute infection. In addition, direct microbiological testing is necessary, and its results should guide the identification of the most appropriate antimicrobial agents. Of note, serological tests require a critical interpretation because unPAD patients may be under immunoglobulin replacement therapy and/or be poorly effective in the early IgM response against specific pathogens [24]. In a recent study by Milito et al., the efficacy of low-dose oral azithromycin prophylaxis (250 mg three times a week for two years) in lowering the incidence of respiratory tract infections was observed in adult primary antibody deficiency (PAD) patients [25]. These results, suggesting the efficacy of antibiotic prophylaxis in PID adults, could also be applied to the pediatric population, but evidence-based data are still missing. The use of cotrimoxazole has been described to prevent the most prevalent bacteria causing respiratory tract infections in patients with reduced serum immunoglobulin levels or immune-mediated conditions [26,27]. However, recent papers did not confirm the efficacy of cotrimoxazole prophylaxis when compared to IRT [28,29]. IRT is the conventional therapy for antibody-deficient inborn errors of immunity [16]. Its efficacy in the control of infection recurrence is well-documented as well as its positive impact on decreasing hospitalizations and mortality rates, ultimately improving patients’ quality of life. IRT in patients with unPAD is not universally recommended, despite some case-series reporting its clinical efficacy [30,31,32,33]. Usually, IRT is considered in those who experience either severe and/or recurrent infections or severe side effects after antibiotic prophylaxis [16]. However, the level of immunoglobulins of unPAD patients considered “protective” is not universally established. The patient’s clinical condition is currently the key factor in orienting the dosage of IRT towards the “individual protective level”. Interestingly, Vivarelli et al. reported the efficacy of low-dose intravenous IRT (0.14 ± 0.06 g/kg/month), administered for one year, in improving serum IgG and IgG subclasses and the annual rate of total infections and hospitalizations in adults with unPAD [34]. It would be interesting to extend this study to the unPAD pediatric population, to dispel the many uncertainties about ITR both in terms of its use and dosage.

UnPAD can be a mutable condition. At the final follow-up examination, 75% of our cohort suffered from a persistent IEI condition, and an unPAD diagnosis was confirmed in fifty percent of cases, whereas the other patients were reclassified as a selective IgM deficiency (16%), CVID (5%), selective IgA deficiency (3%), and IgG3 subclass deficiency (1%). Notably, a retrospective comparison of the clinical and immunological features in the diagnosis of persistent IEI vs. THI cohorts allowed us to identify the following relevant clues of IEI persistence: (I) age at diagnosis > 4 years, (II) history of RRTI and pneumonia, (III) hospitalization due to serious infections and, particularly, to pneumonia, (IV) an isolated or combined IgM deficiency, (V) combined deficiency of IgA and IgM, (VI) combined IgG, IgA, and IgM defect, (VII) low anti-PCP antibody response, and (VIII) low switched memory B cells. We suggest that these clues can be used as “predictive markers” of IEI persistence in the clinical care of unPAD patients. Of note, our preliminary data showed that an asymptomatic status at diagnosis does not represent a potential criterion for Ig normalization over time. Indeed, only in one of the six asymptomatic patients at diagnosis was a THI condition was confirmed. Interestingly, no difference in terms of clinical manifestations at diagnosis has been observed between unPAD and CVID patients at a definitive diagnosis as well as for therapeutic interventions. In fact, among those children requiring IgG replacement therapy or antibiotic prophylaxis at diagnosis, CVID and unPAD were observed in four and six patients, respectively.

Two years ago, a study protocol on unPAD patients was published in the framework of ESID, with the aim to “describe in detail all PAD patients without an identified specific monogenetic defect, [...] support the identification of patients at higher risk for infection or immune dysregulation related complications, enabling in the development of personalized follow-up and treatment plans” [13]. At the conclusion of our analysis, we aim to underline some substantial differences between our study and the study protocol of ESID to dispel any elements of presumed overlap.

Our study enrolls exclusively pediatric patients, whereas the ESID unPAD protocol includes both pediatric and adult patients. Further, the ESID protocol design is to enroll not only unPAD, but all PAD patients who lack a specific monogenetic defect, including a selective IgA deficiency, selective IgM deficiency, and CVID, whereas the IPINeT Ped-unPAD study focuses on the unPAD cohort, as per the previous inclusion criteria. Further, we have also included asymptomatic patients. This choice was based on our previous clinical report that unPAD asymptomatic patients do not necessarily normalize their Ig levels [4]. The IPINeT Ped-UnPAD study has several limitations common to the observational studies. In fact, unPAD patients might not be homogeneously approached due to the paucity of the scientific data and diverse immunological and genetic facilities among the different centers. These limitations could explain missing data in part of the patient cohorts. However, a more extensive inclusion of patients from IPINeT-related centers will allow the standardization of a diagnostic and therapeutic approach for unPAD patients in a national scenario.

## 5. Conclusions

The UnPAD condition is an example of medical complexity. Its heterogeneous clinical phenotype at its onset and variable development of several comorbidities over time strongly indicate that each unPAD patient must be evaluated in their uniqueness, in the aim to develop a tailored therapeutic approach. In this regard, our study might represent a precious source of data to define the natural history and outcome of unPAD patients, laying the groundwork for future research in the field of unPAD-related disorders. It represents a relevant point of reference for the design of therapeutic clinical trials and the elaboration of a diagnostic/therapeutic work-up for unPAD patients in the perspective of personalized/precision medicine. Despite its limitations, a major strength of our study is represented by the more than 20 years of cooperation within the IPINeT Network and with structured international collaborations which might reinforce a shared research project in the field. The early recognition of specific warning signs as well as a multidisciplinary approach, providing the expertise of different medical specialists in collaboration with the immunologist in charge, is essential for the diagnosis and monitoring of unPAD patients. In this scenario, the Ped-unPAD Italian Registry aims to point out the various aspects of the unPAD condition and its phenotypic heterogeneity, representing a powerful resource to support the knowledge and awareness of Health Care Professionals (HCPs), with the aim to provide optimal diagnostic and therapeutic offers for the care of unPAD patients.

## 6. Summary Box

- UnPAD is a heterogeneous and mutable clinical entity, mostly representing a “basket diagnosis”. Long-term monitoring is key to a better understanding of each patient’s long-term health trajectory and for the conduct of a definitive diagnosis.
- UnPAD is usually symptomatic with a wide range of clinical manifestations. A proportion of unPAD patients experience severe infectious and non-infectious manifestations, sometimes requiring hospitalization.
- Asymptomatic UnPAD children deserve accurate monitoring since a diagnostic reclassification might occur. An asymptomatic status at diagnosis may not predict a benign outcome.
- Some UnPAD patients may show abnormal B-cell memory subsets and a variable specific Ab response, as observed in other antibody deficiencies, including CVID.
- A proportion of UnPAD children may evolve into THI. Some patients may maintain the UnPAD status and others may be later reclassified as CVID, a selective IgA deficiency, or a selective IgM deficiency.
- Preliminary data in UnPAD children show the following predictive markers of IEI persistence: (a) age at diagnosis > 4 years, (b) history of RRTI and pneumonia, (c) hospitalization due to severe infections and, particularly, to pneumonia, (d) a combined Ig isotype deficiency, (e) a poor specific Ab response, (f) impaired switched memory B cell subsets.
- In the setting of fever and signs suggestive of an acute infectious process, the prompt and accurate clinical evaluation and initiation of broad-spectrum antimicrobial therapy are required. Additionally, direct microbiological testing is essential, with the subsequent tailoring of antimicrobial therapy based on the cultured organism and its antimicrobial susceptibility profile.
- IRT in patients with unPAD is not universally recommended; however, IRT should be considered in those who experience either severe and/or recurrent infections or severe side effects after antibiotic prophylaxis.
- Considering the risk of both infectious and non-infectious complications, a multidisciplinary approach providing the expertise of different medical specialists in collaboration with the immunologist in charge, is essential to deliver the best possible care to unPAD patients.

## Figures and Tables

**Table 1:** Candidate genes associated with Primary Antibody Deficiencies (according to Bousfiha et al. 2022 [17]).

AICDA	MAP3K14
AKT1	CD20
BLK	MYD88
BLNK	NFKB1
BTK	NFKB2
CD19	IKBA
CD27	IKBB
CD40	IKBE
CD40L	PAX5
CD79A	PIK3CD
CD79B	PIK3R1
CD81	PLCG2
IKKA	PRKCD
CR2	PTEN
CTLA4	RAC2
FYN	REL
ICOS	RELA
IFNGR1	RELB
IFNGR2	STK4
IKKB	SYK
IKZF1	TCF3
IL12B	EVER1
IL12RB1	EVER2
IL21	TNFRSF13B
IL21R	TNFRSF13C
IRAK4	TNFRSF17
IRF2BP2	TNFSF12
ISG15	TNFSF13
LAT	TNFSF13B
LRBA	UNG
LYN	VAV1
IGHM	IGLL1
SLC39A7	TOP2B
SPI1	FNIP1
PIK3CG	ATP6AP1
MOGS	TRNT1
IKZF2	IKZF3
ARHGEF	SH3KBP1
SEC61A1	CTNNBL1
CD21	TWEAK
POU2AF1	MSH6
INO80	IGKC
CARD11	

**Table 2:** Research Interest of Ped-unPAD study.

1. Incidence and minimal prevalence of unPAD in Italian scenario
2. Median age at disease onset and symptoms at onset
3. Median age at diagnosis (time span from clinical onset to clinical diagnosis to quantify diagnostic delay)
4. Clinical–immunological profile at diagnosis and during follow-up
5. Treatment performed and patient’s response
6. Genetic Characterization
7. Quality of Life

**Table 3:** e-CRF Collected Information.

Demographic data	Patient’s initials Date of birth
Family History	Family History (IEI, autoimmunity, allergy, etc.) Consanguinity
Clinical Manifestations	- Absence of symptoms - Age at symptoms onset - Infectious diseases at diagnosis - Etiological agent(s) - Allergic diseases - Non-infectious gastrointestinal disorders - Non-infectious respiratory diseases - Lymphoproliferative Disorders - Neoplasia - Autoimmune Diseases - Hospitalization (causes and durancy)
Treatments	- Previous and current therapies - Ig Replacement Therapy (IVIG, SCIG, and F-SCIG) - Antimicrobial Prophylaxis - Other
Laboratory Investigations	- Complete Blood Count - Immunoglobulins (IgG, IgA, IgM, and sIgE) - IgG Subclasses - s-FLC: Kappa sFLC and Lambda sFLC - Standard and Extended B- and T-Lymphocytes subsets - Specific Ab responses (Anti TT Ab; Anti PCP Ab, etc.) - Autoimmunity (Autoantibodies, C3, C4)
Genetic analysis	Not yet performed, ongoing, or carried out Type of mutation
Instrumental investigations (on clinical indication)	- Spirometry - Abdomen ultrasound - EGDS - Chest CT
Quality of life	Days of absence/year (School/Job) Recreative activities (practiced or not)

**Table 4:** Clinical–immunological findings and therapies of 110 patients with an initial diagnosis of unPAD, according to age at diagnosis (<4 and >4 years).

	unPAD (110 pts)	<4 Years (59 pts)	>4 Years (51 pts)	*p* Value
CLINICAL MANIFESTATIONS	104/110 (94%)	55/59 (93%)	49/51 (96%)	ns
Infections	84/104 (80%)	46/55 (83%)	38/49 (77%)	ns
RRTI	26/84 (31%)	8/46 (17%)	18/38 (47%)	0.04
Otitis	28/84 (33%)	16/46 (35%)	12/38 (32%)	ns
Bronchiolitis	17/84 (20%)	14/46 (30%)	3/38 (8%)	0.01
Bronchitis	11/84 (13%)	6/46 (13%)	5/38 (13%)	ns
Sinusitis	13/84 (15%)	8/46 (17%)	5/38 (13%)	ns
Pneumonia	26/84 (31%)	9/46 (19%)	17/38 (45%)	0.018
Gastroenteritis	14/84 (17%)	11/46 (24%)	3/38 (8%)	ns
Viral Hepatitis	1/84 (1%)	1/46 (2%)	0/38 (0%)	ns
UTI	7/84 (8%)	6/46 (13%)	1/38 (3%)	ns
Meningitis	2/84 (2%)	0/46 (0%)	2/38 (5%)	ns
Skin Abscesses	5/84 (6%)	0/46 (0%)	5/38 (13%)	0.016
Giardiasis	2/84 (2%)	2/46 (4%)	0/38 (0%)	ns
Rheumatic disease	2/84 (2%)	2/46 (4%)	0/38 (0%)	ns
Skin infections	1/84 (1%)	1/46 (2%)	0/38 (0%)	ns
Allergy	37/104 (36%)	14/55 (25%)	23/49 (47%)	0.0257
Food Allergy	8/37 (22%)	4/14 (29%)	4/23 (17%)	ns
Atopic Dermatitis	15/37 (40%)	6/14 (43%)	9/23 (39%)	ns
Rhinitis	23/37 (62%)	9/14 (64%)	14/23 (61%)	ns
Urticaria	5/37 (13%)	2/14 (14%)	3/23 (13%)	ns
Asthma	12/37 (32%)	3/14 (21%)	9/23 (39%)	ns
Conjunctivitis	12/37 (32%)	4/14 (29%)	8/23 (35%)	ns
Angioedema	1/37 (3%)	1/14 (7%)	0/23 (0%)	ns
Vernal keratoconjunctivitis	1/37 (3%)	0/14 (0%)	1/23 (4%)	ns
Autoimmunity	9/104 (9%)	2/55 (4%)	7/49 (14%)	ns
Hashimoto Thyroiditis	1/9 (11%)	1/2 (50%)	0/7 (0%)	ns
Type 1 Diabetes	1/9 (11%)	0/2 (0%)	1/7 (14%)	ns
Psoriasis	1/9 (11%)	0/2 (0%)	1/7 (14%)	ns
Celiac Disease	3/9 (33%)	0/2 (0%)	3/7 (43%)	ns
Alopecia	1/9 (11%)	0/2 (0%)	1/7 (14%)	ns
Nephrotic syndrome	2/9 (22%)	0/2 (0%)	2/7 (29%)	ns
Glomerulonephritis	1/9 (11%)	1/2 (50%)	0/7 (0%)	ns
Non-infective pulmonary diseases	2/104 (2%)	2/55 (4%)	0/49 (0%)	ns
Bronchiectasis	1/2 (50%)	1/2 (50%)	-	-
Cystic Fibrosis	1/2 (50%)	1/2 (50%)	-	-
Benign lymphoproliferation	4/104 (4%)	2/55 (4%)	2/49 (4%)	ns
Lymphadenopathies	3/4 (75%)	2/2 (100%)	1/2 (50%)	ns
Splenomegaly	1/4 (25%)	0/2 (0%)	1/2 (50%)	ns
Hepatomegaly	0/4 (0%)	0/2 (0%)	0/2 (0%)	ns
Neoplasia	0/104 (0%)	-	-	-
HOSPITALIZATION	28/110 (25%)	15/59 (25%)	13/51 (25%)	ns
Pneumonia	10/28 (36%)	2/15 (13%)	8/13 (61%)	0.016
Meningitis	2/28 (7%)	0/15 (0%)	2/13 (15%)	ns
Bronchiolitis	6/28 (21%)	4/15 (27%)	2/13 (15%)	ns
Gastroenteritis	3/28 (11%)	3/15 (20%)	0/13 (0%)	ns
UTI	2/28 (7%)	1/15 (7%)	1/13 (8%)	ns
RRTI	7/28 (25%)	2/15 (13%)	5/13 (38%)	ns
Cellulitis	1/28 (4%)	0/15 (0%)	1/13 (8%)	ns
Mononucleosis	1/28 (4%)	1/15 (7%)	0/13 (0%)	ns
Rheumatic disease	2/28 (7%)	1/15 (7%)	1/13 (8%)	ns
IMMUNOLOGICAL ABNORMALITIES	110/110 (100%)	59/59 (100%)	51/51 (100%)	ns
Isolated or combined IgG defect	81/110 (74%)	45/59 (76%)	36/51 (71%)	ns
Isolated or combined IgA defect	52/110 (47%)	37/59 (63%)	15/51 (29%)	0.0006
Isolated or combined IgM defect	60/110 (54%)	34/59 (58%)	26/51 (51%)	ns
IgG subclass deficiency	59/84 (70%)	33/43 (77%)	26/41 (63%)	ns
Poor specific antibody response to tetanus	8/54 (15%)	4/35 (11%)	4/19 (21%)	ns
Poor specific antibody response to pneumococcus	13/43 (30%)	10/27 (37%)	3/16 (19%)	ns
Low switched memory B cells	22/44 (50%)	13/26 (50%)	6/18 (33%)	ns
Low IgM memory B cells	4/44 (9%)	1/26 (4%)	3/18 (17%)	ns
THERAPIES				
Ig Replacement therapy	3/110 (3%)	2/59 (3%)	1/51 (2%)	ns
Antibiotic Prophylaxis	7/110 (6%)	3/59 (5%)	4/51 (8%)	ns

**Table 5:** Clinical–immunological findings and therapies at diagnosis of 83 persistent IEIs patients vs. 27 THI patients at last FU.

	Persistent IEIs (83 pts)	THI (27 pts)	*p* Value
Consanguinity	2/83 (24%)	0/27 (0%)	ns
Positive Family History for IEIs	11/83 (13%)	3/27 (11%)	ns
Age at diagnosis > 4 years	45/83 (54%)	6/27 (22%)	0.004 (0.002 multivariate)
Clinical manifestations at diagnosis	78/83 (94%)	26/27 (96%)	ns
Infections	61/78 (78%)	23/26 (88%)	ns
RRTI	32/61 (52%)	6/23 (26%)	0.03
Otitis	16/61 (26%)	12/23 (52%)	ns
Bronchiolitis	11/61 (18%)	6/23 (26%)	ns
Bronchitis	5/61 (8%)	2/23 (9%)	ns
Sinusitis	10/61 (16%)	3/23 (13%)	ns
Pneumonia	22/61 (36%)	4/23 (17%)	0.04
Gastroenteritis	10/61 (16%)	4/23 (17%)	ns
Viral Hepatitis	1/61 (2%)	0/27 (0%)	ns
UTI	3/61 (5%)	4/23 (17%)	ns
Meningitis	2/61 (3%)	0/23 (0%)	ns
Skin Abscesses	5/61 (8%)	0/23 (0%)	ns
Giardiasis	2/61 (3%)	0/23 (0%)	ns
Rheumatic Disease	2/61 (3%)	0/23 (0%)	ns
Skin Infections	0/61 (0%)	1/23 (4%)	ns
Allergy	31/78 (39%)	5/26 (19%)	ns
Autoimmunity	8/78 (10%)	1/26 (4%)	ns
Non-infective pulmonary diseases	2/78 (3%)	0/26 (0%)	ns
Benign lymphoproliferation	20/78 (26%)	8/26 (31%)	ns
HOSPITALIZATION	20/78 (26%)	8/26 (31%)	ns
Pneumonia	10/20 (50%)	0/8 (0%)	0.02
Meningitis	2/20 (10%)	0/8 (0%)	ns
Bronchiolitis	2/20 (10%)	4/8 (50%)	0.04
Gastroenteritis	1/20 (5%)	2/8 (25%)	ns
UTI	1/20 (5%)	1/8 (12%)	ns
RRTI	5/20 (25%)	0/8 (0%)	ns
Cellulitis	1/20 (5%)	0/8 (0%)	ns
Mononucleosis	1/20 (5%)	0/8 (0%)	ns
Rheumatic disease	2/20 (10%)	0/8 (0%)	ns
HOSPITALIZATION due to serious infections	13/20 (65%)	1/8 (12%)	0.03
IMMUNOLOGICAL ABNORMALITIES AT DIAGNOSIS	83/83 (100%)	27/27 (100%)	ns
Isolated or combined IgG defect	59/83 (71%)	22/27 (81%)	ns
Isolated or combined IgA defect	42/83 (50%)	10/27 (37%)	ns
Isolated or combined IgM defect	50/83 (60%)	10/27 (37%)	0.046 (0.012 multivariate)
Isolated IgG defect	18/ 83 (22%)	14/27 (52%)	0.0062 (<0.001 multivariate)
Combined IgG, IgA, IgM defect	23/83 (28%)	3/27 (11%)	ns
IgG and IgA defect	10/83 (12%)	3/27 (11%)	ns
IgG and IgM defect	8/83 (10%)	2/27 (7%)	ns
Combined IgA and IgM defect	7/83 (8%)	3/27 (11%)	0.05 (multivariate)
IgM and IgG subclass defect	4/83 (5%)	0/27 (0%)	ns
IgA and IgG subclass defect	3/83 (4%)	1/27 (4%)	ns
IgG subclass deficiency	42/63 (67%)	16/21 (76%)	ns
Poor specific antibody response to tetanus	6/39 (15%)	2/16 (12%)	ns
Poor specific antibody response to pneumococcus	13/31 (42%)	1/12 (8%)	0.04
Low switched memory B cells	19/37 (51%)	0/9 (0%)	0.006
Low IgM memory B cells	4/37 (11%)	0/9 (0%)	ns
THERAPIES	10/78 (13%)	0/26 (0%)	0.054
Ig Replacement therapy	3/78 (4%)	-	-
Antibiotic Prophylaxis	7/78 (9%)	-	-

## Data Availability

The raw data supporting the conclusions of this article will be made available by the authors on request.
